# A mutant vesicular stomatitis virus with reduced cytotoxicity and enhanced anterograde trans-synaptic efficiency

**DOI:** 10.1186/s13041-020-00588-3

**Published:** 2020-03-20

**Authors:** Kunzhang Lin, Xin Zhong, Min Ying, Lei Li, Sijue Tao, Xutao Zhu, Xiaobin He, Fuqiang Xu

**Affiliations:** 1 Wuhan National Laboratory for Optoelectronics, Huazhong University of Science and Technology, Wuhan, 430074 China; 2 Center for Brain Science, State Key Laboratory of Magnetic Resonance and Atomic and Molecular Physics, Key Laboratory of Magnetic Resonance in Biological Systems, Wuhan Center for Magnetic Resonance, Wuhan Institute of Physics and Mathematics, Innovation Academy for Precision Measurement Science and Technology, Chinese Academy of Sciences, Wuhan, 430071 China; 3 University of Chinese Academy of Sciences, Beijing, 100049 PR China; 4 Shenzhen Key Lab of Neuropsychiatric Modulation, Guangdong Provincial Key Laboratory of Brain Connectome and Behavior, CAS Key Laboratory of Brain Connectome and Manipulation, The Brain Cognition and Brain Disease Institute (BCBDI), Shenzhen Institutes of Advanced Technology, Chinese Academy of Sciences, Shenzhen, China; 5 Shenzhen-Hong Kong Institute of Brain Science-Shenzhen Fundamental Research Institutions, Shenzhen, 518055 China; 6 Center for Excellence in Brain Science and Intelligence Technology, Chinese Academy of Sciences, Shanghai, 200031 China

**Keywords:** Neurotropic virus, Output network tracing, Vesicular stomatitis virus, Attenuated cytotoxicity, Enhanced anterograde trans-synaptic tracing

## Abstract

Understanding the connecting structure of brain network is the basis to reveal the principle of the brain function and elucidate the mechanism of brain diseases. Trans-synaptic tracing with neurotropic viruses has become one of the most effective technologies to dissect the neural circuits. Although the retrograde trans-synaptic tracing for analyzing the input neural networks with recombinant rabies and pseudorabies virus has been broadly applied in neuroscience, viral tools for analyzing the output neural networks are still lacking. The recombinant vesicular stomatitis virus (VSV) has been used for the mapping of synaptic outputs. However, several drawbacks, including high neurotoxicity and rapid lethality in experimental animals, hinder its application in long-term studies of the structure and function of neural networks. To overcome these limitations, we generated a recombinant VSV with replication-related N gene mutation, VSV-N_R7A_, and examined its cytotoxicity and efficiency of trans-synaptic spreading. We found that by comparison with the wild-type tracer of VSV, the N_R7A_ mutation endowed the virus lower rate of propagation and cytotoxicity in vitro, as well as significantly reduced neural inflammatory responses in vivo and much longer animal survival when it was injected into the nucleus of the mice brain. Besides, the spreading of the attenuated VSV was delayed when injected into the VTA. Importantly, with the reduced toxicity and extended animal survival, the number of brain regions that was trans-synaptically labeled by the mutant VSV was more than that of the wild-type VSV. These results indicated that the VSV-N_R7A_, could be a promising anterograde tracer that enables researchers to explore more downstream connections of a given brain region, and observe the anatomical structure and the function of the downstream circuits over a longer time window. Our work could provide an improved tool for structural and functional studies of neurocircuit.

## Introduction

Analyzing the connection of brain neural networks, including input and output neural pathways, is the basis of understanding the mechanisms for brain functions and brain diseases [1, 2]. Trans-synaptic tracing technology based on neurotropic viruses is one of the most effective means to characterize brain neural networks, because viral vectors can be used not only to analyze the structural connections of neural networks, but also to express different genes for functional manipulation [3–11].

Mapping brain connectome requires both retrograde and anterograde trans-synaptic neuronal circuit tracers [3, 12, 13]. In retrograde trans-synaptic labeling system, pseudorabies virus (PRV) as a trans-multi-synaptic tracer, is widely used in mapping the connection between peripheral-central and central connections [14, 15]. Rabies virus (RV), a rigorous trans-mono-synaptic tracer, is broadly used to analyze input brain circuits [4]. Other viral strains or mutants, such as CVS-N2c^ΔG^, self-inactivated replication deficient rabies virus (SIRV) and G/L gene deletion rabies virus (RV-ΔGL), which can further reduce toxicity, attain long-term gene manipulation for functional studies [6, 13, 16]. In anterograde trans-synaptic labeling system, herpes simplex virus type 1 (HSV-1) strain 129 (H129) and vesicular stomatitis virus Indiana serotype (VSV-Ind) are efficient anterograde trans-multi-synaptic tracers for the analysis of output networks [7, 17]. H129, a DNA virus with complex genome, can be used for anterograde multi-trans-synaptic pathways from type-specific starter cells [8]. H129-ΔTK-tdT, with AAV virus complementarily expressing thymidine kinase gene (TK), has been reported for anterograde trans-mono-synaptic tracing to visualize direct projections from a specific neuron type [10]. Vesicular stomatitis virus (VSV) has the advantages of simpler structure, higher expression of exogenous genes and wider infection range [7, 18–20]. Envelope glycoprotein of VSV can be replaced by glycoproteins of other viruses to achieve different labeling characteristics and direction controllable trans-synaptic transfer [21, 22]. But these VSVs have high cytotoxicity that can lead to rapid death of mice, which limits long-term structural observation and gene manipulation or function research, restricting their applications in neuroscience [23]. Several mutants have been obtained by mutating the N gene related to the replication of VSV [24–27]. After mutating the seventh amino acid of the N protein (VSV-N_R7A_), the replication speed of the virus decreased by 2–3 orders of magnitude within 24 h [24, 25], which might be used as a better tracer, but whether it still has the ability of anterograde trans-synaptic labeling is still unknown.

Here, we examined the features of VSV-N_R7A_ as an anterograde trans-synaptic tracer. We found that VSV-N_R7A_ exhibits attenuated cytotoxicity, delayed but more efficient anterograde trans-synaptic capability. We conclude that VSV-N_R7A_ can provide longer time window for neural circuits research, making it a possible new tool for the study of brain output networks.

## Materials and methods

### Construction of the recombinant mutant VSV vector

pVSV-EGFP was bought from Addgene (plasmid # 36399, Connie Cepko’s lab). To construct the mutant VSV (VSV-N_R7A_), N gene with the seventh amino acid mutation (R to A) was amplified and inserted into the pVSV-EGFP digested by restriction enzyme MscI and BSTZ17I (New England Biolabs). The sequences of primers designed to amplify N mutant were: N′(R7A)-F: tggccaTATGAAAAAAACTAACAGTAATCAAAATGTCTGTTACAGTCAAGGCCATC; N′(R7A)-F1: ATCATTGACAACACAGTCATAG; N′(R7A)-F2: ATGTCTGTTACAGTCAAGGCCATCATTGACAACACAGTCATAG; N′(R7A)-R: gtatacTCAATGTCATCAGGCTGTCGGGCATT. Plasmids were verified by DNA sequencing.

### Rescue and preparation of recombinant VSV

Rescue of wild-type VSV (WT-VSV) and mutant VSV (Mutant-VSV) is as previously reported [28]. BHK-21 cells with 6-well plate were incubated with vaccinia carrying T7 RNA polymerase for 1 h, then washed with PBS three times, and co-transfected with VSV genome plasmid and packaging plasmids encoding the N, P and L proteins in certain proportions. After 6 h, the supernatant was discarded, and 2 ML Dulbecco’s minimum essential media (DMEM) containing 5% fetal bovine serum (FBS) was added to the 6-well plate and cultured in 31 °C, 5% (v/v) CO2 incubator. After 120 h, the supernatant was collected and filtered with 0.1 μm filter to infect normal BHK-21 cells. Cells were observed by inverted fluorescence microscopy (Olympus) 24 h later. The supernatants were collected after complete lesion, filtered with 0.22 μm membrane and centrifuged at 50000×g for 2.5 h at 4 °C. The precipitation was suspended with PBS and then concentrated and purified with 20% sucrose for the second time. The precipitation was suspended with appropriate amount of PBS. The titer was determined by plaque assay and the virus was stored at − 80 °C.

### Single-step growth curves of viruses

Single-step growth curves were carried out at 35 °C to determine the replication efficiency of the VSVs. BHK-21 cells were grown in twelve-well plates to a density of 70%~ 80%, and infected with each recombinant virus at a multiplicity of infection (MOI) of 3. After 1 h of incubation at 35 °C, the viral inoculum was removed, cells were washed 3 times with phosphate buffered saline (PBS), and 1.5 ml of fresh DMEM medium (containing 3% FBS) was added. Supernatants were harvested at different time points (12, 24, 36, 48, 72 and 96 h post infection (hpi)), and virus titers were detected by plaque assay.

### Virus injection and slices preparation

In animal experiments, all surgical and experimental procedures were carried out in accordance with the guidelines formulated by the Animal Care and Use Committee of Wuhan Institute of Physics and Mathematics, Chinese Academy of Sciences, and experiments related to VSV were performed in Biosafety Level-2 (BSL-2) laboratory. Eight-week-old C57BL/6 mice (20–25 g) were used for VSV injection, and injection process was referred to previous study [29]. 0.1 ul 5 × 10^8^ FFU/ml recombinant VSV was injected into VTA and DG area of dorsal hippocampus. The stereotactic coordinates for DG were: AP: − 2.00 mm; ML: 1.00 mm; DV: − 2.00 mm from the bregma. For VTA were: AP: − 3.20 mm; ML: 0.45 mm; DV: − 4.30 mm from the bregma. After a certain day, the mice were anesthetized with 5% chloral hydrate (600 mg/kg), and perfused with 0.9% saline and 4% polyformaldehyde solution respectively. Then, the brains were soaked overnight in 4% paraformaldehyde solution. After dehydration was completed with 30% sucrose solution, the brain was sectioned with the thickness of 40 μm by frozen section machine. Brain slices were washed 3 times with PBS, 5 min each time. After DAPI staining for 10 min, the brain slices were attached to the microscope slides and sealed with 70% glycerol.

### Immunohistochemistry and fluorescence imaging

For staining neurons in vivo, the target brain slices were sealed with 10% sheep serum for 1.5 h, and immunostained with a Cy3-conjugated rabbit antibody against NeuN (diluted by PBS at 1:300, Merck Millipore, ABN78C3) overnight at 4 °C. For staining microglia cells in vivo, brain slices were blocked with 10% sheep serum in PBS with 0.3% Triton X-100 for 1.5 h, then incubated with primary antibody (anti-Iba1: diluted by PBS at 1:1000, LAK4357, WAKO) overnight at 4 °C. After washed 3 times with PBS, slices were incubated with the secondary antibody cy3-conjugated goat anti-rabbit immunoglobulin G (IgG) (1:400, 94,600, Jackson) for 1 h at 37 °C. All the brain slices above were washed 3 times with PBS, then attached to the microscope slides and sealed with 70% glycerol. Imaging was performed by using the Olympus VS120 Slide Scanner microscope (Olympus).

## Results

### Mutant-VSV can effectively infect neurons in vivo

**Fig. 1:**
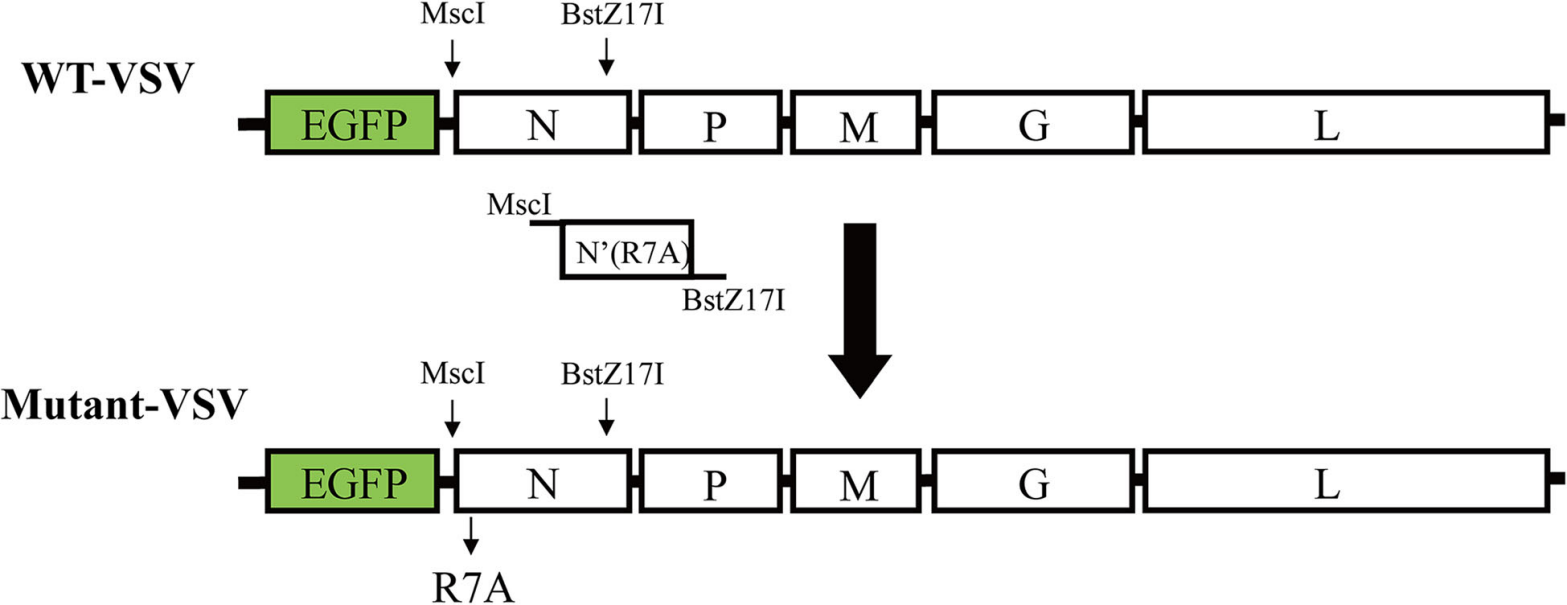
Construction of recombinant VSV with mutant N gene. To construct the recombinant mutant VSV vector, N gene with the seventh amino acid mutation (R to A) was amplified and inserted into the pVSV-EGFP digested by restriction enzyme MscI and BSTZ17I

**Fig. 2:**
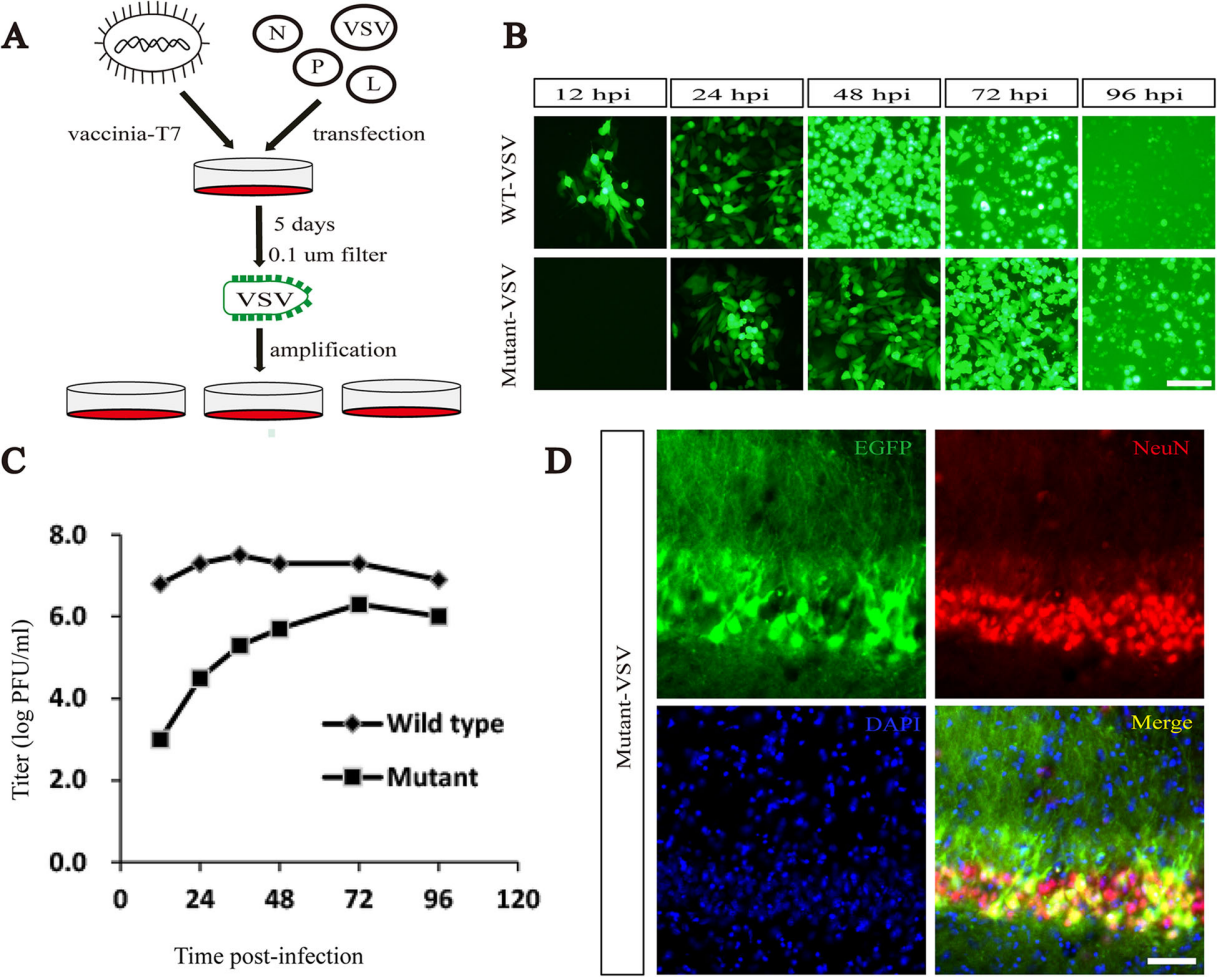
Mutant-VSV effectively infects neurons in vivo. **a** Rescue and preparation process of VSV. BHK-21 cells were pretreated with vaccinia carrying T7 RNA polymerase for one hour, then washed with PBS three times, and co-transfected with VSV genome plasmid and packaging plasmids encoding the N, P and L proteins. After 6 h, they were replaced with fresh culture medium and placed in a 31 °C incubator. 5 days later, the supernatant was collected and filtered with 0.1 μm filter membrane to remove the vaccinia, then added to BHK-21 cells to amplify the VSV. **b** Fluorescent imaging of BHK-21 cells infected with WT-VSV and Mutant-VSV at an MOI of 0.001. When infected with WT-VSV, obvious fluorescence signals can be detected within 12 h, while Mutant-VSV needs 24 h. **c** The single-step growth curves of the WT-VSV and Mutant-VSV. The viruses were collected and titered on BHK-21 cells at indicated time points (12, 24, 36, 48, 72 and 96 hpi). **d** Fluorescent signals from mutant VSV were co-localized with neurons in vivo. Cell nuclei were stained using DAPI (blue), neurons were stained with Cy3-conjugated NeuN antibody. Scale bars =100 μm for B and D

Based on the characteristics of rapid amplification, high infection efficiency and expression level of exogenous genes, VSV has been developed into an important gene transfer vector [7, 19]. It has been previously reported that rearrangement or mutation of viral genes in the genome can reduce the replication speed or toxicity [30–34]. Here, recombinant VSV-N_R7A_ was constructed (Fig. 1). The wild-type and mutant VSV with EGFP reporter were recovered and amplified in BHK21 cells (Fig. 2a). WT-VSV and Mutant-VSV were added to BHK-21 cells at an MOI (multiplicity of infection) of 0.001. When infected with WT-VSV, obvious fluorescence signals can be detected within 12 h, while Mutant-VSV need 24 h (Fig. 2b). Obvious cytopathic changes were observed after 48 h in WT-VSV group, however, cytopathies were observed 72 h later in VSV-N_R7A_ group (Fig. 2b). Compared to WT-VSV, Mutant-VSV exhibits a reduction (three orders of magnitude) in virus replication at 35 °C within 24 hpi (Fig. 2c), which is consistent with previous findings [24]. In addition, the titer of Mutant-VSV increased with time and reached its peak in 72 h, which was one order of magnitude less than that of wild-type VSV (Fig. 2c). The ability of the Mutant-VSV to infect neurons in vivo was investigated. The Mutant-VSV was injected into the DG region of mouse brain. The brain slices were prepared at 1 day post-injection (DPI) and stained with Cy3-conjugated NeuN antibody. Results showed that EGFP fluorescent signals from Mutant-VSV were co-localized with neurons in vivo (Fig. 2d).

### Neuroinflammatory responses induced by the WT-VSV and mutant-VSV vectors

**Fig. 3:**
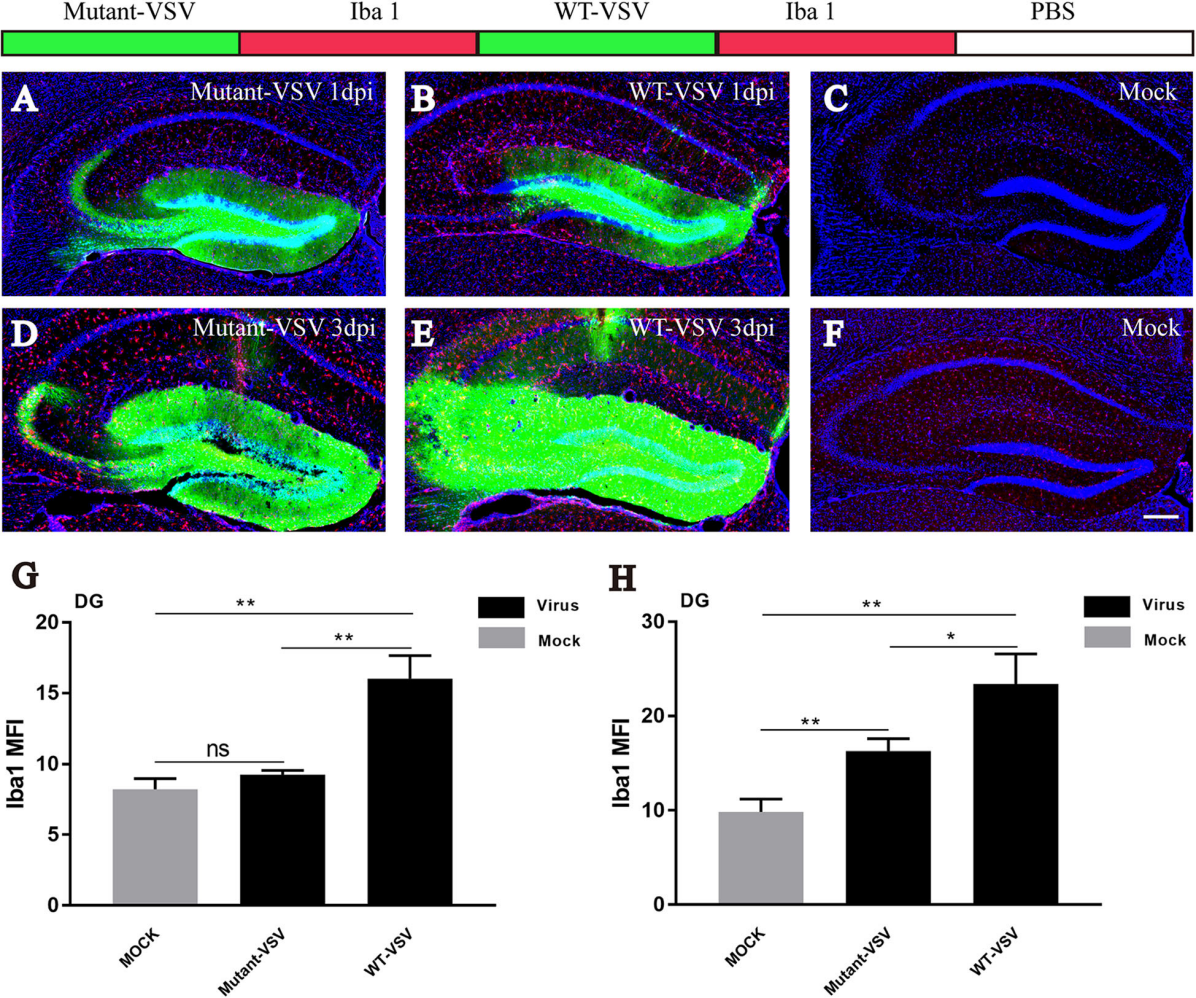
Analysis of inflammatory responses induced by WT-VSV and Mutant-VSV at injection site. **a**-**c** Immunostaining for the microglial marker Iba1 at injection site. Iba1-positive cells were observed in the WT-VSV group at 1 DPI, but barely in the Mutant-VSV or PBS injected (Mock) DG. **d**-**f** Intense microglial activation was observed at the injection site when WT-VSV or Mutant-VSV was injected into the DG at 3 DPI, and signals in the WT-VSV group were more than that in the Mutant-VSV group, while few signals were observed in the PBS injected (Mock) DG. **g** Quantification of mean fluorescence intensity (Mean ± SEM) of Iba1 in DG at 1 DPI. Mutant-VSV: 9.251 ± 0.182, WT-VSV: 16.02 ± 0.9505, Mock: 8.22 ± 0.4402, Mutant-VSV vs Mock: *P* = 0.0963; WT-VSV vs Mock: *P* = 0.0017; WT-VSV vs Mutant-VSV: *P* = 0.0022; *n* = 3. **h** Quantification of mean fluorescence intensity (Mean ± SEM) of Iba1 in DG at 3 DPI. Mutant-VSV: 16.29 ± 0.7438, WT-VSV: 23.41 ± 1.836, Mock: 9.828 ± 0.7976, Mutant-VSV vs Mock: *P* = 0.0041; WT-VSV vs Mock: *P* = 0.0025; WT-VSV vs Mutant-VSV: *P* = 0.0229; n = 3. n is mice number. Scale bars = 200 μm for A-F. Unpaired t-tests, *P < 0.05, **P < 0.01, ***P < 0.001

Viral infections can cause neuroinflammation and induce microglia activation [35, 36]. To evaluate microglial activation induced by WT-VSV and Mutant-VSV infection at the injection site, immunohistochemistry for Iba1, the microglial marker, was performed. Obvious Iba1-positive cells were observed in the WT-VSV group at 1 DPI, but barely in the Mutant-VSV or PBS injected (Mock) DG (Fig. 3a-c). Intense microglial activation was observed at the injection site when WT-VSV or Mutant-VSV was injected into the DG at 3 DPI, and signals in the WT-VSV group were more than that in the Mutant-VSV group, while few signals were observed in the PBS injected DG (Fig. 3d-f). By quantification of mean fluorescence intensity (Mean ± SEM) of Iba1 in DG at 1 DPI (Fig. 3g, Mutant-VSV: 9.251 ± 0.182, WT-VSV: 16.02 ± 0.9505, Mock: 8.22 ± 0.4402, Mutant-VSV vs Mock: *P* = 0.0963; WT-VSV vs Mock: *P* = 0.0017; WT-VSV vs Mutant-VSV: *P* = 0.0022) and 3 DPI (Fig. 3h, Mutant-VSV: 16.29 ± 0.7438, WT-VSV: 23.41 ± 1.836, Mock: 9.828 ± 0.7976, Mutant-VSV vs Mock: *P* = 0.0041; WT-VSV vs Mock: *P* = 0.0025; WT-VSV vs Mutant-VSV: *P* = 0.0229), we found that microglial infiltration was markedly increased at the injection site (DG region) following WT-VSV injection, while microglial activation due to Mutant-VSV infection was significantly milder even after 3 days (Fig. 3). These results indicated that Mutant-VSV was less toxic in the injection site.

### Anterograde trans-synaptic labeling with mutant-VSV vector

**Fig. 4:**
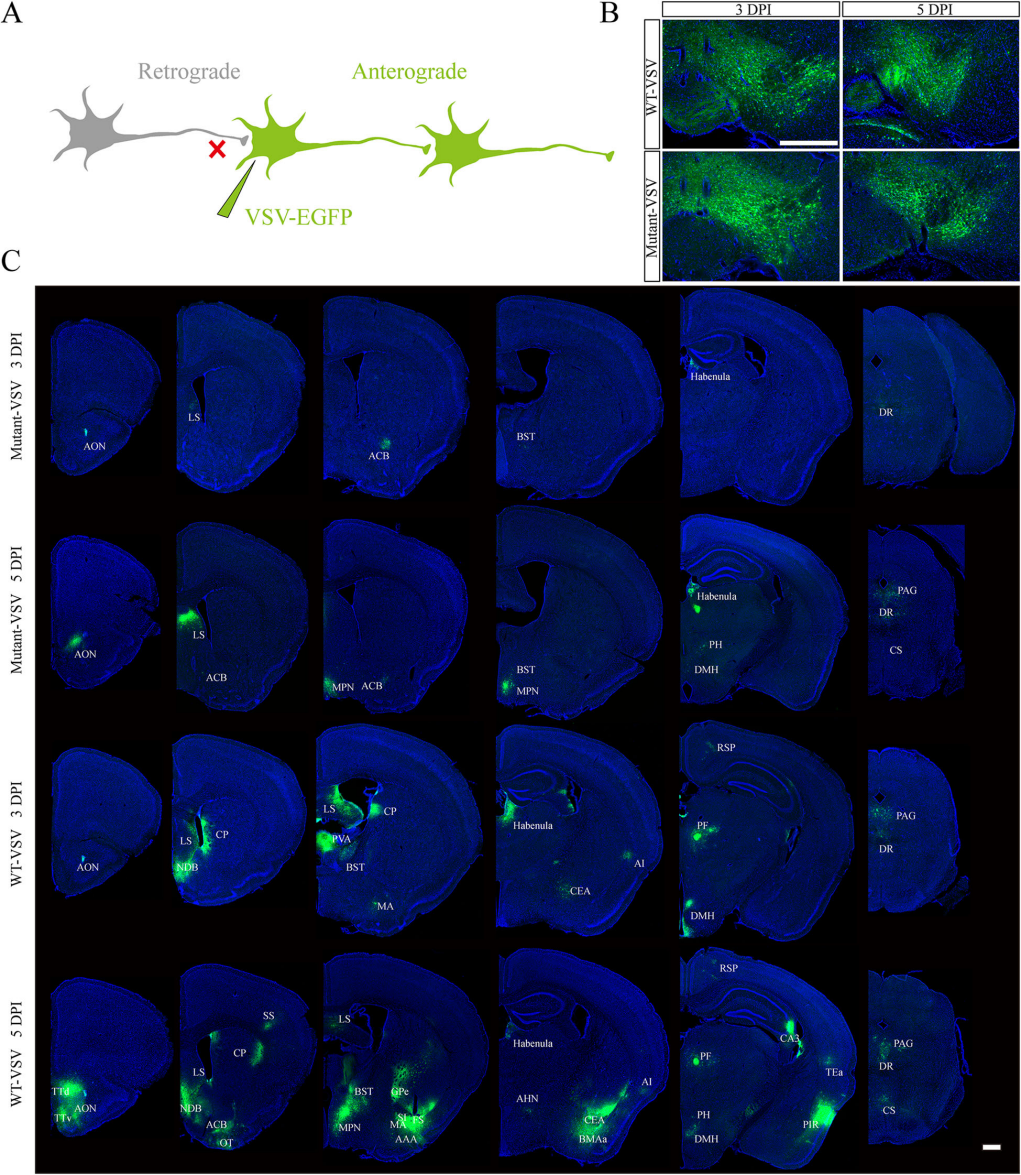
Anterograde trans-synaptic labeling with Mutant-VSV after injection into the VTA. **a** Schematic of recombinant VSV anterograde trans-synaptic labeling. **b** The brain slices of injection site at indicated time points (3 DPI and 5 DPI) were imaged. **c** Anterograde trans-synaptic labeling with Mutant-VSV at indicated time points. Mutant VSV can effectively infect downstream brain area of VTA, and more brain regions were labeled over time, but less brain regions were labeled by Mutant-VSV than by WT-VSV at 5 DPI, which showed that Mutant-VSV had delayed anterograde trans-synaptic ability. The EGFP signal was imaged by using the Olympus VS120 Slide Scanner microscope. Scale bars =500 μm for B and C. The specific name of the brain areas labeled were shown as follows: AON: Anterior olfactory nucleus; TTd: Taenia tecta, dorsal part; TTv: Taenia tecta, ventral part; LS: Lateral septal nucleus; ACB: Nucleus accumbens; NDB: Diagonal band nucleus; OT: Olfactory tubercle; CP: Caudoputamen; SS: Somatosensory areas; MPN: Medial preoptic nucleus; BST: Bed nuclei of the stria terminalis; GPe: Globus pallidus, external segment; SI: Substantia innominata; FS: Fundus of striatum; MA: Magnocellular nucleus; AAA: Anterior amygdalar area; Habenula: Habenula; AHN: Anterior hypothalamic nucleus; CEA: Central amygdalar nucleus; BMAa: Basomedial amygdalar nucleus, anterior part; AI: Agranular insular area; PH: Posterior hypothalamic nucleus; DMH: Dorsomedial nucleus of the hypothalamus; PF: Parafascicular nucleus; RSP: Retrosplenial area; TEa: Temporal association areas; PIR: Piriform area; CA3: Ammon’s horn Field CA3; DR: Dorsal nucleus raphe; PAG: Periaqueductal gray; CS: Superior central nucleus raphe

VSV has the ability of anterograde trans-synaptic tracing of neural circuits (Fig. 4a), and is used for the output network analysis of specific brain region [37]. Ventral tegmental area (VTA) is an important brain region with complex output neural networks, which performs diverse functions in reward, cognition, motivation, and aversion [38–40]. VTA neurons project to the lateral septal nucleus (LS), the nucleus accumbens (ACB), the caudoputamen (CP), the habenula, the amygdala, the dorsal nucleus raphe (DR), the prefrontal cortex (PFC), the anterior cingulate area (ACA), and the somatomotor areas (MO), among other regions [41–46]. To examine anterograde trans-synaptic ability of Mutant-VSV, the Mutant-VSV and WT-VSV were injected into the VTA region of mouse brain, and the brain slices were prepared at 3 DPI and 5 DPI. Green signals were observed in injection site (Fig. 4b). Several brain regions including anterior olfactory nucleus (AON), lateral septal nucleus (LS), nucleus accumbens (ACB), bed nuclei of the stria terminalis (BST), habenula, and dorsal nucleus raphe (DR) were labeled by Mutant-VSV at 3 DPI. However, signals were detected in additional brain regions at 5 DPI, including medial preoptic nucleus (MPN), posterior hypothalamic nucleus (PH), dorsomedial nucleus of the hypothalamus (DMH), periaqueductal gray (PAG), superior central nucleus raphe (CS) (Fig. 4c). Except for MPN, all of these labelled regions received the direct projection from VTA neurons according to Allen Mouse Brain Connectivity Atlas (http://connectivity.brain-map.org/). These results showed that Mutant-VSV has anterograde trans-synaptic ability, and the trans-synaptic efficiency increases with time. Moreover, Mutant-VSV may anterogradely label the postsynaptic neurons through one synaptic connection at 3 DPI.

Moreover, more brain regions were labeled by wild-type VSV at 3DPI and 5 DPI (Fig. 4c), especially at 5 DPI, including taenia tecta, dorsal part (TTd), diagonal band nucleus (NDB), olfactory tubercle (OT), caudoputamen (CP), somatosensory areas (SS), globus pallidus, external segment (GPe), substantia innominata (SI), fundus of striatum (FS), magnocellular nucleus (MA), anterior amygdalar area (AAA), anterior hypothalamic nucleus (AHN), central amygdalar nucleus (CEA), basomedial amygdalar nucleus, anterior part (BMAa), retrosplenial area (RSP), parafascicular nucleus (PF), ammon's horn Field CA3 (CA3), temporal association areas (TEa), and piriform area (PIR). These results showed that the speed of the anterograde trans-synaptic tracing of Mutant-VSV was slowed down.

### Attenuated lethality of mutant-VSV in mice brain compared with WT-VSV

**Fig. 5:**
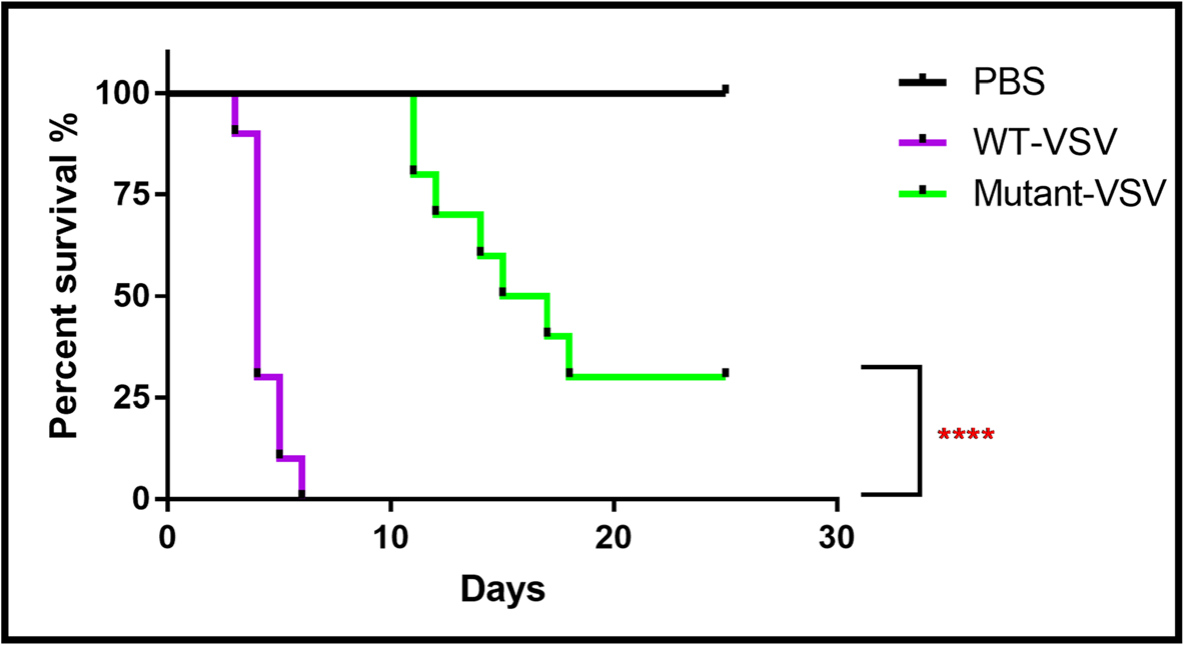
Survival rate of WT-VSV and Mutant-VSV infected mice. WT-VSV, Mutant-VSV and PBS were injected intracranially into the DG domain of adult mice. WT-VSV was rapidly lethal within 1 week and more than half of all deaths occured at 4 DPI, which occured at 14 DPI in Mutant-VSV group. The percent survival was analyzed by Log-rank test (*n* = 10 in each group; *P* < 0.0001). *P < 0.05, **P < 0.01, ***P < 0.001, ****P < 0.0001

Rapid lethality in experimental animals is a limitation for most neurotropic viruses in neuroscience applications [47]. It is important to determine the survival time of mice infected with neurotropic virus, because it can provide technical support for virus application in vivo, such as controlling the time window of infection. Eight-week-old C57BL/6 mice were divided into three groups with 10 mice in each. WT-VSV, Mutant-VSV and PBS were injected intracranially into the DG of hippocampus. WT-VSV was rapidly lethal to experimental animals at 3 DPI and more than half of the deaths occurred at 4 DPI, however, in Mutant-VSV group, the first case of death of experimental animals occurred at 11 dpi, and time of more than half of the deaths was delayed to 15 DPI (Fig. 5). The survival percentage was analyzed by Log-rank test (*P* < 0.0001, Fig. 5). These data showed that as compared with WT-VSV, the time of death in Mutant-VSV infected mice was delayed significantly.

### More connected brain networks were labeled with mutant-VSV

**Fig. 6:**
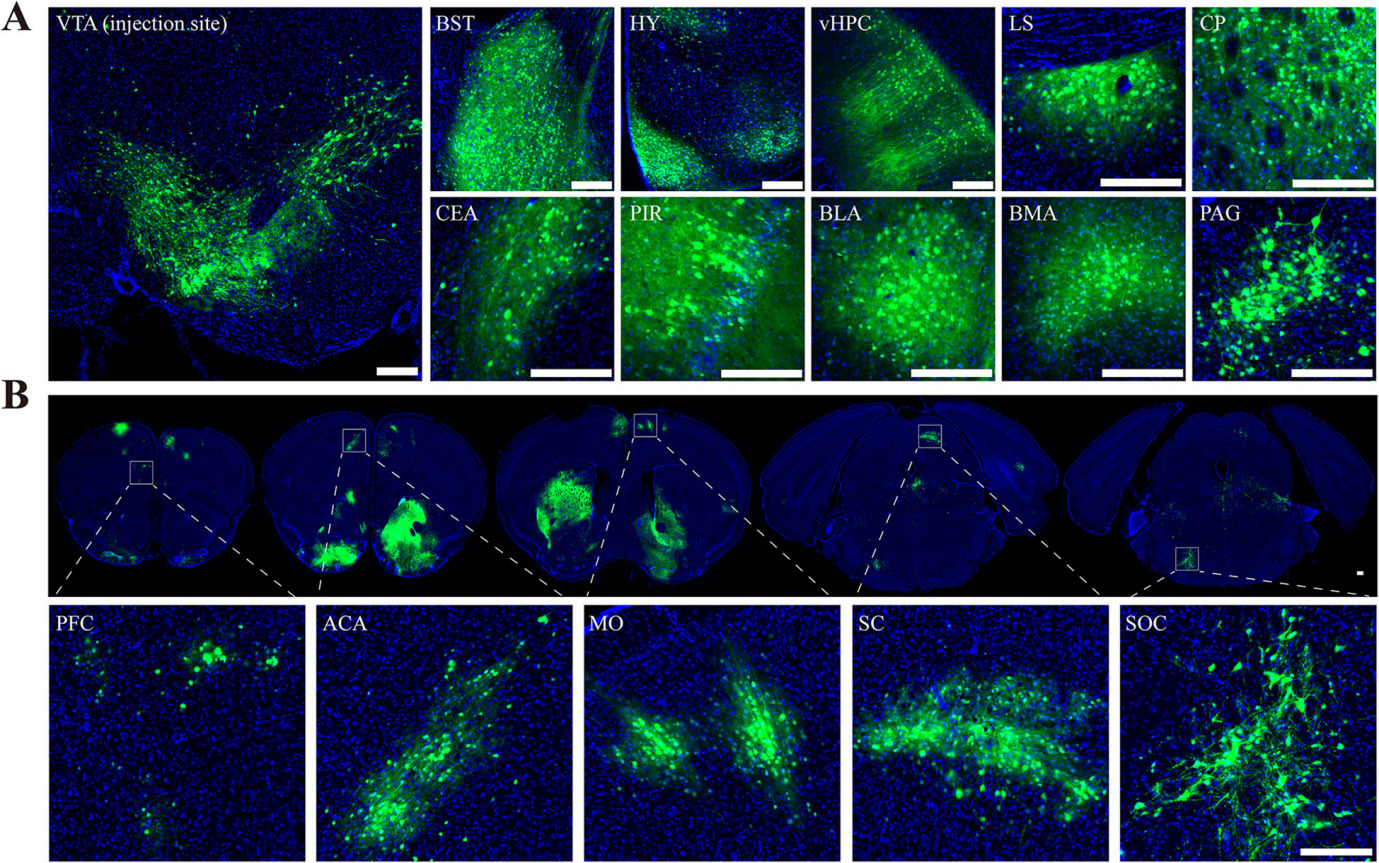
Efficiency of Mutant-VSV in tracing VTA output pathway at 10 days post infection. **a** Normal cell morphology of labeled VTA output neurons can be observed at 10 DPI by using Mutant-VSV. Mutant-VSV were injected into the VTA of C57BL/6 mice, and the brain slices were imaged at 10 days post-injection. **b** More connected downstream brain regions can be revealed through Mutant-VSV. HY: Hypothalamus; vHPC: ventral hippocampus; BLA: Basolateral amygdalar nucleus; PFC: Prefrontal cortex; ACA: Anterior cingulate area; MO: Somatomotor areas; SC: Superior colliculus; SOC: Superior olivary complex. Scale bars =200 μm for A and B

As the rapid death of experimental animals induced by neurotropic virus, the connected brain networks of injection site were not fully resolved. To determine whether the attenuated virus could label more connected network with the extension of survival time, Mutant-VSV were injected into the VTA of C57BL/6 mice, and the brain slices were imaged at 10 days post-injection. Normal cell morphology of labeled VTA output neurons can be observed at 10 DPI by using Mutant-VSV (Fig. 6a). Moreover, additional labeled brain regions, including prefrontal cortex (PFC), anterior cingulate area (ACA), somatomotor areas (MO), superior colliculus (SC) and superior olivary complex (SOC), were observed with Mutant-VSV at 10 DPI (Fig. 6b), which were not labeled by WT-VSV at 5 DPI (Fig. 4c). More complete comparison of the labeled regions under different conditions were shown in Table 1. These results suggested that Mutant-VSV could be used to reveal longer range of downstream networks compared with WT-VSV.

**Table 1:** Assessment of viral spread from WT-VSV and Mutant-VSV injections into the VTA

Brain region	WT-VSV		Mutant-VSV		
	3 DPI	5 DPI	3 DPI	5 DPI	10 DPI
AON	+	++	+	++	++
LS	+	++	+	++	++
ACB (NAc)	+	++	+	+	++++
CP	++	++	–	–	++++
OT	–	+	–	–	+++
BST	+	++	+	+	++++
Habenula	++	+++	+	++	++
Amygdala	+	++	–	–	+++
DR	+	++	+	+	++
PIR	–	++	–	–	++
PFC	–	–	–	–	+
ACA	–	–	–	–	++
MO	–	–	–	–	++
SC	–	–	–	–	++
SOC	–	–	–	–	++

The absence or presence of labeling was indicated by (−) and (+), respectively. The extent of labeling was indicated with the number of (+). Results showed that with the reduced toxicity and extended animal survival, more connected downstream networks were labeled by the Mutant-VSV compared with the WT-VSV

## Discussion

Neural network abnormalities can cause many neurological and psychiatric diseases, such as Parkinson’s disease, Schizophrenia, Alzheimer’s disease and Autism [2]. Analyzing the connection of brain neural networks is the basis of understanding the mechanism of network variation of brain diseases. Neurotropic virus mediated anterograde trans-synaptic tracing is an important approach for mapping the output of brain circuits. Several viral tracers for anterogradely trans-synaptic tracing had been developed [20, 48], but the rapid toxicity and lethality to the infected neurons and animals limited their application in many areas of research that need dissecting the output network according to connection order within a longer time window, which were important to understand not only the anatomical mechanism of the given brain region, but also the variation of the circuits on various neurological and psychiatric disease. In order to overcome these defects, we introduced a replication-related mutation within the nucleoprotein, N_R7A_, into the genome of the VSV. We found that compared with the WT-VSV, the N_R7A_ mutation endowed the virus lower rate of propagation and cytotoxicity in vitro, as well as significantly reduced neural inflammatory responses in vivo and much longer animal survival when it was injected into the nucleus of the mice brain. Besides, the Mutant-VSV exhibited effectively anterograde transsynaptic spread in vivo when injected into the VTA, and the order of labeled connection increased with the extension of infection time. Importantly, with the reduced toxicity and extended animal survival, more connected downstream networks were labeled by the Mutant-VSV compared with the WT-VSV.

Viral toxicity is mainly related to viral replication, virulent genes and host immune response [27, 49, 50]. It has been reported that the toxicity of neurotropic viruses can be reduced by decreasing the replication rate [6, 16]. The N protein binds to the genomic RNA of VSV to form N-RNA complex, which serves as the template for genomic replication and transcription by the RNA-dependent RNA polymerase [51]. The R7A mutation in N protein was reported to reduce the genomic replication and transcription at 37 °C [27]. As expected, by comparison with WT-VSV, Mutant-VSV exhibited a reduction in virus replication and cytotoxicity (Fig. 2 and Fig. 3), which is consistent with previous findings. However, the lower replication rate may affect the expression and trans-synaptic transmission of neurotropic viruses [16]. Therefore, whether it retains the ability of transsynaptic tracing shoud be evaluated. When injected into the VTA region, Mutant-VSV can effectively transmit to the downstream networks at 3 DPI and 5 DPI, although the speed of spreading was slowed down (Fig. 4). Further order of the downstream networks, including prefrontal cortex (PFC), anterior cingulate area (ACA), somatomotor areas (MO), superior colliculus (SC) and superior olivary complex (SOC), were labeled by Mutant-VSV at 10 DPI than that by WT-VSV at 5 DPI, when injected into the VTA of C57BL/6 mice (Fig. 6). Thus, we provide a useful trans-multisynaptic tracer for researchers to dissect downstream neural circuit more efficiently.

With reduced cytotoxicity, the Mutant-VSV makes it possible to deliver optogenetic and calcium indicators to monitor or manipulate the network activity over a longer experimental window. With the canonical anterograde tracer, such as WT-VSV and HSV, the time window that ensures effectively trans-synaptic tracing has been reported to be between 3 and 5 days from the infection [7, 48]. With Mutant-VSV, the useful temporal window for imaging can reach up to 10–15 days from the infection, which overcoming the shortage of relative short survival time of experimental animals. Meanwhile, VSV is a negative-stranded RNA virus, HSV is a double stranded DNA virus, there are many different characteristics between them, including genome, toxicity and application, etc. [52]. We have provided a more detailed comparison (pros and cons) between VSV-N_R7A_ and the more commonly used HSV variants (H129 and H8). A more detailed comparison was shown in Table 2.

**Table 2:** Comparison of viral tracers of anterograde trans-mulisynaptic tracing

Strain	Genome	Dir	Cell toxicity	Days of survival	Expressing level	Operation risk	Cre/flp dependent
**WT-VSV**	-ss RNA 12 kb	A	High	3–5	High	Low	N
**VSV-N(R7A)**	-ss RNA 12 kb	A	Medium	10–15	High	Low	N
**HSV H129**	ds DNA 153 kb	A/R	High	3–5	Medium	Medium	Y
**HSV H8**	ds DNA 153 kb	A/R	High	3–5	High	Medium	Y

Abbreviations: *-ss RNA* Negative-strand, single strand RNA, *ds DNA* Double strand DNA, *Dir* Direction of transsynaptic tracing, “A” Anterograde, “R” Retrograde, “N” Can’t be used as a cre/flp dependent anterograde trans-multisynaptic viral tracer, “Y” Can be used as a cre/flp dependent anterograde trans-multisynaptic viral tracer. VSV is a negative-stranded RNA virus, HSV is a double stranded DNA virus, there are many different characteristics between them, including genome, toxicity and application, etc. Researchers should choose the virus according to different experimental needs

The mutant-VSV, with effective transsynaptic tracing and extended animal survival, also has the potential to achieve time-dependent anterograde trans-synaptic labeling. For example, Mutant-VSV vector that expresses a slow fluorescent timer (sFT), whose fluorescence color changed from blue to red over time, would enable us to differentiate 2nd-order neurons from 1st-order neurons according to the red-to-blue fluorescence intensity ratio [53]. Moreover, Zheng et al. have reported that neural connections can be detected with ex vivo MRI using a ferritin-encoding VSV, but wild-type viral toxicity affects long-term signal acquisition [11]. Therefore, the Mutant-VSV would be an appropriate vector. However, the Mutant-VSV still have some cytotoxicity with the extension of infection time. Future studies are required to further ameliorate the speed of toxicity by using VSV mutants and variants. Because the M protein is one of the main components of VSV’s rapid cytotoxicity by blocking host transcription and RNA export, M mutants (M51R and △M51) have been chose as good candidates to delay toxicity [54–56]. We have developed one such VSV with N_R7A_ and △M51 mutant, VSV-N_R7A_-△M51, and are in the process of evaluating the cytotoxicity and transmission features in vivo.

In summary, our findings suggest that Mutant-VSV (VSV-N_R7A_) can be used as an efficient anterograde trans-multi-synaptic tracer, with attenuated cytotoxicity, and delayed but enhanced anterograde trans-synaptic spreading. The work can provide a promising anterograde tracer that enables researchers to explore more downstream connections of a given brain region, and observe the anatomical structure and the function of the downstream circuits over a longer time window.

## Data Availability

The datasets used or analysed during the current study are available from the corresponding author on reasonable request.

## Abbreviations

VSV: Vesicular stomatitis virus
MOI: Multiplicity of infection
DMEM: DULBECCO’S minimum essential media
PBS: Phosphate buffer saline
DAPI: 4′,6-diamidino-2-phenylindole
DG: Dentate gyrus
VTA: Ventral tegmental area
AON: Anterior olfactory nucleus
TTd: Taenia tecta, dorsal part
TTv: Taenia tecta, ventral part
LS: Lateral septal nucleus
ACB: Nucleus accumbens
NDB: Diagonal band nucleus
OT: Olfactory tubercle
CP: Caudoputamen
SS: Somatosensory areas
MPN: Medial preoptic nucleus
BST: Bed nuclei of the stria terminalis
GPe: Globus pallidus, external segment
SI: Substantia innominata
FS: Fundus of striatum
MA: Magnocellular nucleus
AAA: Anterior amygdalar area
Habenula: Habenula
AHN: Anterior hypothalamic nucleus
CEA: Central amygdalar nucleus
BMAa: Basomedial amygdalar nucleus, anterior part
AI: Agranular insular area
PH: Posterior hypothalamic nucleus
DMH: Dorsomedial nucleus of the hypothalamus
PF: Parafascicular nucleus
RSP: Retrosplenial area
TEa: Temporal association areas
PIR: Piriform area
CA3: Ammon’s horn Field CA3
DR: Dorsal nucleus raphe
PAG: Periaqueductal gray
CS: Superior central nucleus raphe
HY: Hypothalamus
vHPC: ventral hippocampus
BLA: Basolateral amygdalar nucleus
PFC: Prefrontal cortex
ACA: Anterior cingulate area
MO: Somatomotor areas
SC: Superior colliculus
SOC: Superior olivary complex
